# Predictive and Prognostic Value of Oncogene Mutations and Microsatellite Instability in Locally-Advanced Rectal Cancer Treated with Neoadjuvant Radiation-Based Therapy: A Systematic Review and Meta-Analysis

**DOI:** 10.3390/cancers15051469

**Published:** 2023-02-25

**Authors:** Elena De Mattia, Jerry Polesel, Silvia Mezzalira, Elisa Palazzari, Sara Pollesel, Giuseppe Toffoli, Erika Cecchin

**Affiliations:** 1 Experimental and Clinical Pharmacology, Centro di Riferimento Oncologico di Aviano (CRO) IRCCS, Via Franco Gallini n. 2, 33081 Aviano, Italy; 2 Unit of Cancer Epidemiology, Centro di Riferimento Oncologico di Aviano (CRO) IRCCS, Via Franco Gallini n. 2, 33081 Aviano, Italy; 3 Radiation Oncology, Centro di Riferimento Oncologico di Aviano (CRO) IRCCS, Via Franco Gallini n. 2, 33081 Aviano, Italy; 4 Surgical Oncology, Centro di Riferimento Oncologico di Aviano (CRO) IRCCS, Via Franco Gallini n. 2, 33081 Aviano, Italy

**Keywords:** locally advanced rectal cancer, KRAS, MSI, pathological complete response, neoadjuvant chemoradiotherapy

## Abstract

**Simple Summary:**

Identification of novel molecular markers of pathological complete response (pCR) to preoperative radiation-based therapy in locally advanced rectal cancer (LARC) is strongly needed. Given the established predictive and/or prognostic role of somatic mutations in key oncogenes (*RAS, TP53, BRAF, PIK3CA, SMAD4*) and microsatellite instability (MSI) status in colorectal cancer, we aimed to investigate the clinical value of the same markers in LARC patients by systematically reviewing the published literature and performing a quantitative analysis of the data. We found that *KRAS* mutations were significantly associated with the risk of not achieving pCR after preoperative treatment. This association was even more significant in patients who did not receive cetuximab than in patients who did. No other markers were associated with pCR. Based on our results, the implementation of *KRAS* mutation testing into clinical practice could improve the management of LARC patients.

**Abstract:**

Markers of pathological complete response (pCR) to preoperative radiation-based therapy in locally advanced rectal cancer (LARC) are strongly needed. This meta-analysis aimed at elucidating the predictive/prognostic role of tumor markers in LARC. We systematically reviewed the impact of *RAS*, *TP53, BRAF*, *PIK3CA*, and *SMAD4* mutations and MSI status on response (pCR, downstaging) and prognosis (risk of recurrence, survival) in LARC according to PRISMA guidelines and the PICO model. PubMed, Cochrane Library, and Web of Science Core Collection were systematically searched to identify relevant studies published before October 2022. *KRAS* mutations were significantly associated with the risk of not achieving pCR after preoperative treatment (summary OR = 1.80, 95% CI: 1.23–2.64). This association was even more significant in patients not receiving cetuximab (summary OR = 2.17, 95% CI: 1.41–3.33) than in patients receiving cetuximab (summary OR = 0.89, 95% CI: 0.39–20.05). MSI status was not associated with pCR (summary OR = 0.80, 95% CI: 0.41–1.57). No effect of *KRAS* mutation or MSI status on downstaging was detected. Meta-analysis of survival outcomes was not possible due to the large heterogeneity among studies in endpoint assessment. The minimum number of eligible studies to assess the predictive/prognostic role of *TP53*, *BRAF*, *PIK3CA*, and *SMAD4* mutations was not reached. *KRAS* mutation, but not MSI status, proved to be a detrimental marker for response to preoperative radiation-based therapy in LARC. Translating this finding into the clinic could improve the management of LARC patients. More data are needed to clarify the clinical impact of *TP53*, *BRAF*, *PIK3CA*, and *SMAD4* mutations.

## 1. Introduction

Colorectal cancer (CRC) is one of the most commonly diagnosed cancers and one of the leading causes of cancer-related death worldwide [1]. Rectal cancers account for approximately 30–35% of all colorectal cancers, and about half of them are diagnosed at a locally advanced stage (i.e., locally advanced rectal cancer, LARC) [2]. A combined modality approach involving the use of fluoropyrimidine-based neoadjuvant chemoradiotherapy (nCRT), followed by total mesorectal surgical excision, is the standard of care for LARC patients [3,4]. At the time of surgery, a variable proportion of patients (8–30%) achieve pathologic complete response (pCR) [5,6], a condition associated with favorable long-term outcome [7], and with the possibility of opting for an organ-preserving approach (i.e., local excision or watch-and-wait strategy) [8]. On the other hand, early identification of patients with a poor response could be helpful in selecting patients for intensified pre-operative chemotherapy (e.g., total neoadjuvant therapy, TNT) [3,9].

Currently, the baseline assessment of patients for treatment planning is mainly based on clinical-pathological criteria [10] and has only recently been integrated by specific radiomic features [11].

Nowadays, testing for somatic tumor mutations in *RAS* and *BRAF* is mandatory to select the most appropriate treatment for CRC patients, and characterization of microsatellite instability (MSI) status of CRC tumor is assessed to evaluate the use of immune checkpoint inhibitors [12,13]. Although there is increasing evidence of the potential role of these predictive/prognostic molecular markers in LARC, they are still not included in risk algorithms used in clinics.

The mechanism of oncogenesis and the spectrum of molecular changes in tumor tissue have been described as significantly different between colon and rectal tumors [14,15,16]. It has been reported that 82% of non-metastatic rectal cancers have mutations in cancer-driving genes belonging to the PI3K and MAPK pathways, including *KRAS*, *PIK3CA*, and *TP53* [17], similar to colon cancer, but the distribution of these mutations was different between rectal and colon samples [14,16]. For example, TP53 pathway mutations were more common in rectal tumors, whereas colon carcinomas had more RAS and PI3K pathway alterations [14,16]. The pharmacogenomic role of *KRAS* and *TP53* mutations has been extensively studied in LARC patients treated with nCRT [18], but their predictive and prognostic value in this setting remains uncertain due to the large heterogeneity of published data. Other genes that have been studied in rectal cancer with contrasting results include *BRAF*, *PIK3CA*, *SMAD4*, and tumor MSI [18,19].

The aim of the present study was to perform a systematic review and meta-analysis of the impact of somatic mutations in the *RAS* (i.e., *KRAS*, *NRAS*, *HRAS*), *TP53*, *BRAF*, *PIK3CA*, and *SMAD4* genes, and MSI status on response to neoadjuvant radiation-based therapy in patients with LARC. The primary endpoints were pCR and tumor downstaging and the secondary endpoints were disease-free survival (DFS) or relapse-free survival (RFS) and overall survival (OS). Determining the true predictive/prognostic value of somatic alterations in LARC could support their application in the clinic to improve selection of the most appropriate therapeutic option.

## 2. Materials and Methods

### 2.1. Literature Search

The systematic review was conducted according to the PRISMA (Preferred Reporting Items for Systematic Reviews and Meta-Analyses) guidelines and the PICO (Patients, Interventions, Comparisons, Outcomes) model (see Supplementary Methods for details on the PICO framework). This review was not registered on PROSPERO.

The literature search was performed for all studies published related to candidate gene mutation/MSI status and its impact on response to neoadjuvant chemotherapy or radiotherapy in LARC patients. Three databases, MEDLINE (PubMed), Cochrane Library, and Web of Science Core Collection (Clarivate), were used to search for relevant articles published in English in a peer-reviewed journal with the last search update on 1 October 2022. Because MEDLINE included all articles found in the other two databases, only MEDLINE was used. Search algorithms included all keywords to indicate ‘rectal cancer’, ‘chemoradiotherapy’ or ‘radiotherapy’, and ‘candidate gene’ or ‘MSI’, combined using Boolean operators (OR/AND) (see Supplementary Methods for the exact literature search algorithm). Additional studies were identified by hand-searching the references of relevant articles. Retrieved articles were screened and selected by two independent authors (EDM and SM) on the basis of inclusion and exclusion criteria; disagreements were resolved by a third researcher (EC). When studies overlapped, data from the publication with the largest number of patients were considered.

### 2.2. Inclusion and Exclusion Criteria

Inclusion criteria were as follows: (1) studies of patients diagnosed with primary adenocarcinoma of the rectum by pathological confirmation and eligible for preoperative radiation-based treatment. Patients with disease at all stages (I–IV) were included for analysis of response (i.e., pCR and downstaging), whereas only patients with stage II–III disease were considered for analysis of DFS/RFS and OS; (2) studies of patients treated with neoadjuvant chemoradiotherapy or radiotherapy; (3) studies that assessed the impact of mutational status in at least one candidate gene (i.e., *RAS, TP53, BRAF, PIK3CA,* and *SMAD4*) or MSI profile on clinical outcome (i.e., pathologic response as assessed by tumor regression grade, downstaging, recurrence, and survival) after neoadjuvant chemoradiotherapy or radiotherapy; (4) studies that obtained molecular data from pre-treatment tumor tissue sample; and (5) studies reporting pCR by mutation/MSI status or odds ratio (OR) with 95% confidence interval (CI). Exclusion criteria included: (1) reviews, meta-analyses, case reports, and conference abstracts; (2) preclinical studies; (3) studies without clinicopathologic endpoints; (4) studies not related to locally advanced rectal cancer; (5) studies with unclear, inadequate, insufficient, or incomplete data; and (6) studies that obtained molecular data from post-treatment biological samples to exclude the effects of chemoradiotherapy on tumor biology (e.g., induced change in the mutational pattern or complete disappearance of tumor cells in samples from patients with a pCR); and (7) case selection or treatment bias.

### 2.3. Data Extraction and Outcomes

Based on inclusion/exclusion criteria, relevant data were extracted in duplicate from all eligible studies by two independent investigators (EDM and SM) and recorded in a dedicated form designed at the beginning of the study. Particularly, the following items were collected for descriptive purposes: first author name, year of publication, country of origin, total number of patients, mean/median age of patients, gender, study type (retrospective/prospective), enrollment interval, disease stage, median follow-up, neoadjuvant treatment, type of chemotherapy, radiotherapy dose, interval time to surgery, type of surgery, and adjuvant treatment information, clinical outcome evaluated, tumor regression grade (TRG) classification system for evaluating pathologic response (i.e., American Joint Commission on Cancer—AJCC [20], Japanese Society for Cancer of the Colon and Rectum —JSCCR [21], Gavioli et al. [22], Dworak et al. [23], Wheeler et al. [24], Ryan et al. [25]), molecular markers studied and the related analytical method, source of biological sample. Discrepancies were resolved by discussion. The number of pCR by mutational/MSI status and the OR with corresponding CI—when available—were retrieved for the statistical analysis.

In this study, the outcomes collected include pCR, downstaging, DFS/RFS, and OS.

### 2.4. Quality Assessment

The methodological quality of each article included in the present meta-analysis was independently assessed by two authors using the Newcastle–Ottawa Scale (NOS) [26] (The Newcastle–Ottawa Scale, 2022), with scores ranging from 0 to 9. A NOS score of ≥7 was considered high quality.

### 2.5. Statistical Analysis

Summary estimates of the proportion of pCR or OR with the corresponding 95% CI were calculated when three or more studies were available. ORs were retrieved from the original publications, when available, or derived from the absolute number of patients with pCR by mutational/MSI status. The random-effects models by DerSimonian and Laird [27] were applied to incorporate both within- and between-study variability, as a weighted average, giving each study a weight proportional to its precision using the logit transformation. Statistical heterogeneity between studies was assessed using the *I*^2^ and τ^2^ statistics [27]. Influence analysis was performed when the summary estimate was estimated from five or more studies: the summary estimate was calculated by omitting one study at a time. Publication bias was assessed using a funnel plot [28]. Considering the existing gene/drug interaction between *KRAS* and cetuximab, analyses for *KRAS* status were stratified by cetuximab neo-adjuvant treatment. Results of the meta-analysis were presented graphically using forest plots, plotting the estimates from individual papers, summary estimate, proportion, and 95% CI. Statistical significance was claimed for *p* < 0.05. Analyses were performed using R’ software.

## 3. Results

### 3.1. Eligible Studies

**Figure 1:**
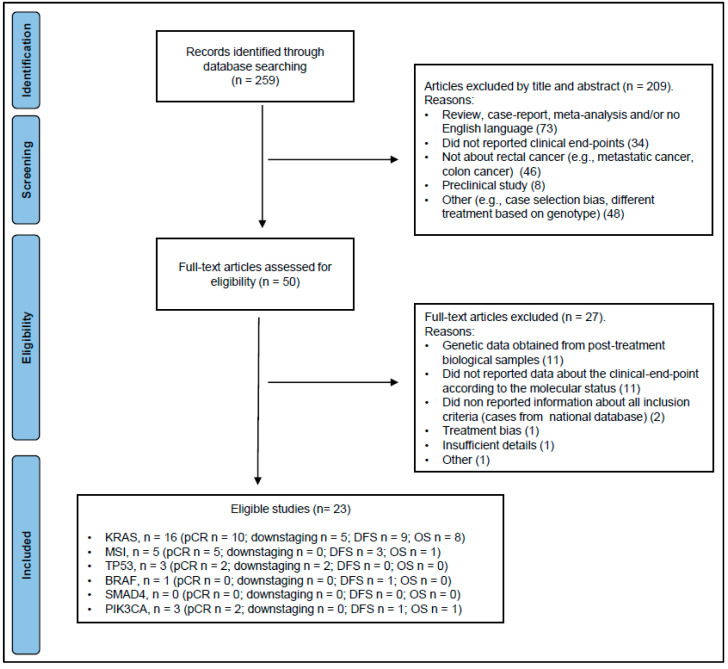
PRISMA flow diagram. Abbreviations: DFS, disease-free survival; OS, overall survival; pCR, pathologic complete response.

Figure 1 summarizes the results of the literature search.

By applying the inclusion/exclusion criteria, the minimum number of three eligible articles required for quantitative synthesis of data was not reached for the *TP53, BRAF, PIK3CA*, and *SMAD4* genes for both response (i.e., pCR and tumor downstaging) and prognosis (i.e., DFS/RFS, OS) endpoints.

By applying the inclusion/exclusion criteria, sufficient articles were found for *RAS* genes and MSI status to perform a quantitative synthesis of the data for the pCR endpoint. For *RAS* genes, tumor downstaging was also analyzed. For DFS/RFS and OS, quantitative analysis was not possible due to large heterogeneity in endpoint assessment and data presentation (e.g., assessment of local or distant recurrence, different follow-up, different statistical methods); therefore, only descriptive synthesis was reported. Regarding the *RAS* genes, only *KRAS* was considered in the present meta-analysis, as no eligible records were identified for the other members of the *RAS* family, with the exception of two articles that investigated *NRAS* mutations [29,30].

### 3.2. KRAS

#### 3.2.1. Pathological Complete Response

Ten eligible studies [29,31,32,33,34,35,36,37,38,39] were considered for quantitative synthesis (Figure 1). Among others, a large study on LARC cases extracted from the national oncologic outcome database was excluded due to the large heterogeneity of the study population, a lack of information on all inclusion criteria, and the risk of replicated data [40]. The characteristics of the included studies and details on the method of molecular analysis and response assessment are provided in Table S1. A summary of the main features of the studies is presented in Table 1. The total number of patients included in the analysis was 965, with an incidence of *KRAS* gene mutation of 35.0% (338/965) and a percentage of pCR of 19.8% (191/965). Seven of the ten eligible studies were conducted in European or North American populations. Most of the studies utilized long course radiotherapy delivered over 5 weeks. Preoperative treatment regimens varied, and the interval to surgery ranged from 0 to 61.4 weeks. The mutational status of *KRAS* was determined mainly by sequencing-based methods (8 of the 10 studies), and exon 2 (i.e., codons 12 and 13) was the most frequently studied. The quality of the included studies was high, with a NOS score equal to 7 for all articles (Table S2).

**Figure 2:**
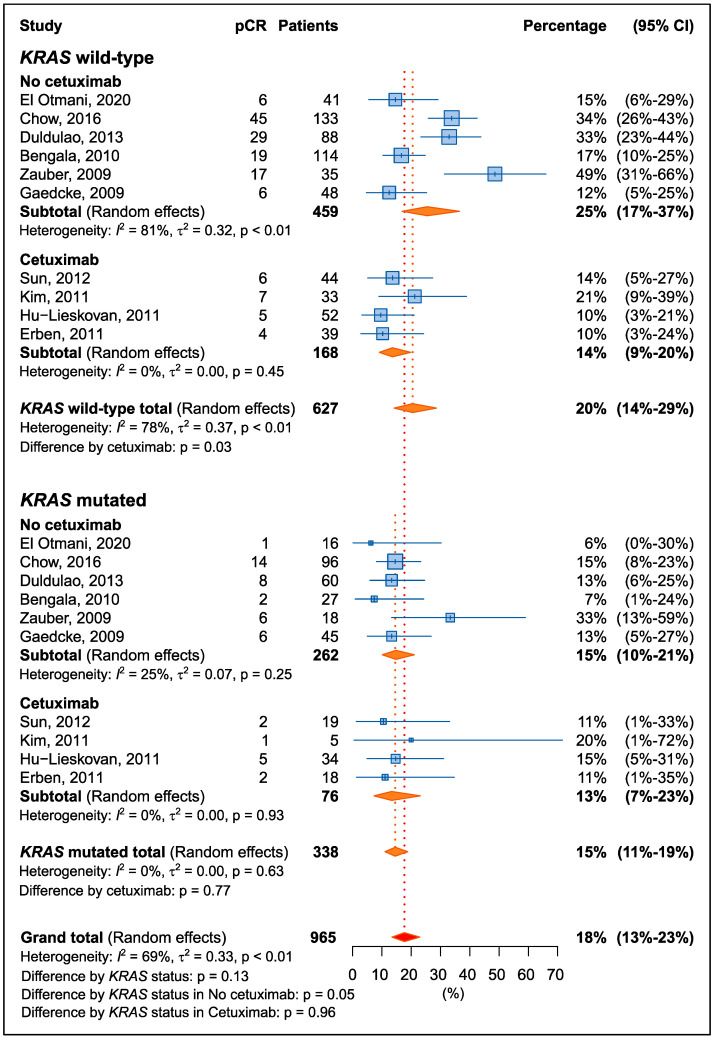
Forrest plot for percentage of pathological complete response (pCR) by *KRAS* mutation and cetuximab treatment [29,31,32,33,34,35,36,37,38,39].

Overall, patients with a *KRAS* mutation have a non-significant lower rate of pCR compared to patients with wild-type *KRAS* (15% and 20%, respectively; *p* = 0.13) (Figure 2). However, treatment with cetuximab had a significant impact on pCR, particularly in patients with wild-type *KRAS* (*p* = 0.03). When analyses were restricted to patients not treated with cetuximab, patients with the *KRAS* mutation had worse pCR (15%; 95% CI: 10–21%) than wild-type patients (25%; 95% CI: 17–37%; *p* = 0.05). There was no difference in patients treated with cetuximab by *KRAS* status (*p* = 0.96).

**Figure 3:**
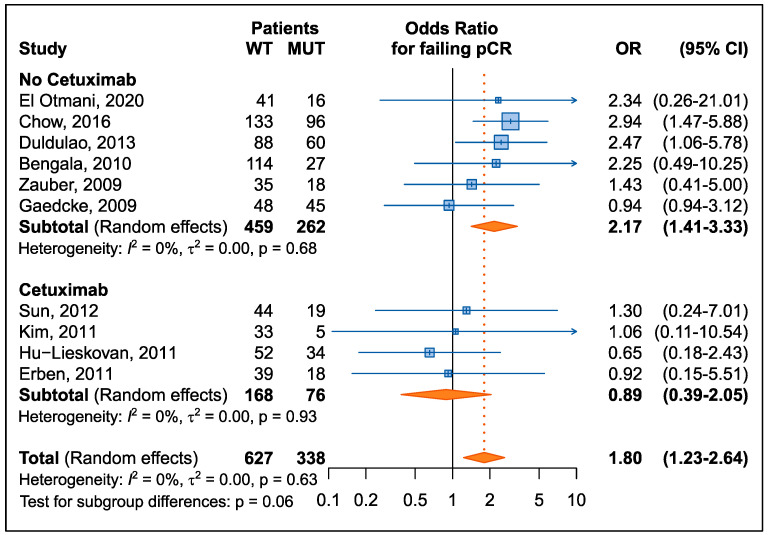
Forrest plot for the risk of not achieving a pathological complete response (pCR) in patients with a *KRAS* mutation according by cetuximab treatment [29,31,32,33,34,35,36,37,38,39].

To account for heterogeneity between studies, the effect of *KRAS* mutational status on the risk of not achieving pCR was expressed as OR for each study (Figure 3). The results showed that the presence of a *KRAS* mutation was significantly associated with an increased risk of not achieving pCR (summary OR = 1.80, 95% CI: 1.23–2.64); no heterogeneity was observed (*p* = 0.63). An analysis stratified by cetuximab use confirmed previous findings: the risk of not achieving pCR was 2.17 (95% CI: 1.41–3.33) in patients not treated with cetuximab compared to 0.89 (95% CI: 0.39–2.05) in patients treated with cetuximab; however, this difference was only marginally significant (*p* = 0.06).

#### 3.2.2. Downstaging

A total of five eligible studies [31,34,35,38,41] that reported data on *KRAS* mutations and their effects on downstaging were considered for data extraction (Figure 1). Four articles [31,35,38,41] analyzed tumor downstaging, three [34,35,41] analyzed T-downstaging, and one [41] analyzed N-downstaging (Table 2). No significant association was found with *KRAS* mutation.

#### 3.2.3. Recurrence Risk

Nine studies [29,30,34,35,37,38,41,42,43] were identified that investigated the impact of *KRAS* mutations on recurrence risk (i.e., DFS, RFS) (Figure 1). However, quantitative analysis of the data could not be performed due to the large heterogeneity of the studies in terms of the method of endpoint assessment (e.g., evaluation of local or distant recurrence, different follow-up, different statistical methods). In summary, eight of nine eligible studies [29,34,35,37,38,41,42,43] showed no association between tumor *KRAS* mutation status and DFS or RFS. Only one study [30] indicated that patients with *KRAS* mutations had a lower 3-year DFS (68% vs. 88.3%, *p* = 0.016) than patients without *KRAS* mutations. Of note, El Otmani et al. [29], in a subgroup analysis according to the specific codon mutations of *KRAS*, showed a significant association between mutations detected at codon 146 (i.e., A146T and A146V) and the presence of both recurrence and distant metastases (*p* = 0.019).

#### 3.2.4. Overall Survival

Eight articles [29,30,35,38,40,41,42,44] examining the effects of *KRAS* mutations on OS were eligible (Figure 1). However, quantitative analysis of the data could not be performed because of the large heterogeneity in the clinical assessment of the endpoint (e.g., different follow-up, different statistical methods). In brief, five studies [29,35,38,41,42] found no significant association between *KRAS* mutations and OS. On the contrary, three studies [30,40,44] reported that patients with *KRAS* mutations had a worse prognosis and an increased risk of death.

### 3.3. MSI Status

#### 3.3.1. Pathological Complete Response

Five eligible studies [29,39,45,46,47] were included in this analysis (Figure 1). Mismatch repair deficiency (dMMR) was classified as high-frequency MSI (MSI-H), whereas proficient mismatch repair (pMMR) or low-frequency MSI (MSI-L) was considered microsatellite stable (MSS) [48]. Among others, two large studies that used a national oncologic outcome database to select LARC cases were excluded due to the large heterogeneity of the study population, lack of information on all inclusion criteria, and risk of replicated data [40,49]. A recent article was also excluded due to treatment bias, as patients received the immune checkpoint inhibitor nivolumab, whose efficacy is known to be related to MSI status [50]. The characteristics of the studies included in the meta-analysis, as well as details on the method of molecular analysis and response assessment, are provided in Table S3. A summary of the main features of the studies can be found in Table 3. The total number of patients included in the analysis was 613, the incidence of MSI-H was 11.7% (72/613), and the percentage of pCR was 15.2% (93/613). The studies were performed in different countries. Preoperative treatment regimens varied, and the interval time to surgery ranged from 3 to 16 weeks. MSI status was determined by immunohistochemistry in three articles and by an allelic size analysis in two articles. The quality of the included studies was high, with a NOS score equal to 7 for all but one article, which had a score of 9 (Table S2).

**Figure 4:**
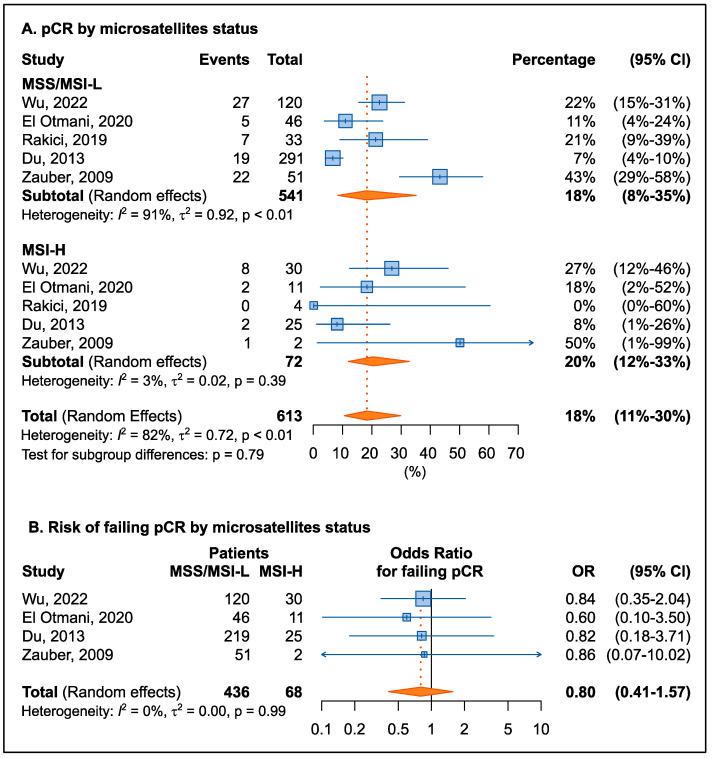
Forrest plot for percentage of pathological complete response (pCR, (**A**)) and for the risk of not achieving a pCR (**B**) by microsatellite status. dMMR was considered as MSI-H, while pMMR or MSI-L are considered as microsatellite stable MSS [29,39,45,46,47].

Microsatellite status (MSI-H or MSI-L/MMS) was not associated with pCR rate (20% vs. 18% for MSI-H and MSI-L/MSS, respectively) (Figure 4A). To control heterogeneity between studies, OR was calculated for each study (Figure 4B). The results confirmed that there was no association between microsatellite status and risk of not responding to therapy (summary OR = 0.80, 95% CI: 0.41–1.57). It should be noted, however, that all studies consistently reported a nonsignificant trend toward a lower risk of non-response to therapy for carriers of tumor MSI-H status.

#### 3.3.2. Downstaging

No eligible article was found that reported data on the association between MSI status and tumor downstaging (Figure 1).

#### 3.3.3. Recurrence Risk

Three eligible studies [29,45,46] were identified that examined the role of MSI status on recurrence risk (i.e., DFS, RFS) (Figure 1). In all studies, no significant association was found between the molecular marker and clinical outcome. Notably, Du et al. [45], in a subgroup analysis performed by tumor stage, showed that in the ypN0 group, patients with MSI-H had significantly better DFS than those with MSI-L or MSS status (100% vs. 79.8%, *p* < 0.05), whereas in the ypN + group no DFS improvement was observed for patients with MSI-H.

#### 3.3.4. Overall Survival

Only one eligible article [29] (Figure 1) examined the impact of MSI status on OS and showed no significant association.

### 3.4. TP53, BRAF, PIK3CA, and SMAD4

#### 3.4.1. Pathological Complete Response

According to the inclusion/exclusion criteria for study selection applied here, no eligible study was identified for the *BRAF* and *SMAD4* genes evaluating the role of mutations on pCR (Figure 1).

For the *TP53* gene, two eligible articles were retrieved [32,51]. The prospective study by Lopez-Crapez et al. [51], which included 70 LARC patients treated with preoperative radiotherapy or chemoradiotherapy, failed to demonstrate an association between *TP53* mutations and pCR. Similarly, the retrospective analysis by Chow et al. [32] of 229 tumor biopsies from LARC patients who received nCRT confirmed that the presence of *TP53* mutations did not affect pCR. For *PIK3CA* gene, two eligible retrospective studies were identified [52,53]. The study by Abdul-Jalil et al. [52] on 201 LARC biopsy specimens from patients treated with nCRT showed that mutations in the *PIK3CA* gene tended to be associated with a lack of pCR (OR: 3.33; *p* = 0.094). In contrast, the work of Russo et al. [53] in 47 LARC patients who had received nCRT showed no association between *PIK3CA* mutations and pCR.

#### 3.4.2. Downstaging

For the *BRAF, PIK3CA,* and *SMAD4* genes, no eligible studies were found that investigated the role of genetic tumor mutations on tumor downstaging (Figure 1).

For the *TP53* gene, two eligible articles were found [51,54]. In the retrospective study by Kandioler et al. [54], which included 64 LARC patients treated with preoperative short-term radiotherapy, it was reported that *TP53* mutations were significantly associated with no response to radiotherapy (*p* < 0.005) in terms of T-downstaging. However, this finding was not confirmed by the prospective analysis of Lopez-Crapez et al. [51], which was performed on 70 LARC patients treated with preoperative radiotherapy or chemoradiotherapy and found no association between *TP53* mutational status and T-downstaging.

#### 3.4.3. Recurrence Risk

For the *TP53, BRAF,* and *SMAD4* genes, no eligible articles evaluating the role of mutations on recurrence risk were found (Figure 1).

For the *PIK3CA* gene, only one eligible study was identified [30]. This retrospective work by Peng et al., involving 70 LARC patients treated with preoperative chemoradiotherapy, found no association between *PIK3CA* mutations and 3-years DFS (3-year rate, 68.6% vs. 82.8% for mutated and wild-type patients, respectively, *p* = 0.632).

#### 3.4.4. Overall Survival

For the *TP53*, *BRAF,* and *SMAD4* genes, no eligible papers were identified examining the association between mutations and survival (Figure 1).

For the *PIK3CA* gene, only one eligible study was found [30]. This retrospective work by Peng et al., including 70 LARC patients treated with nCRT, found no association between *PIK3CA* mutations and 3-years OS (3-year rate, 77.8% versus 94.9% for mutated and wild-type patients, respectively, *p* = 0.870).

### 3.5. Sensitivity Analysis and Publication Bias

Influence analysis was conducted by performing meta-analysis, excluding one study at a time. The results were stable for the estimation of the pCR proportion according to *KRAS* status (Figure S1A,B). Conversely, substantial variability was observed when analyzed by microsatellite stability: for MSS/MSI-L (Figure S1C), the pCR proportion ranged from 14% excluding the study by Zauber et al. [39] to 24%, excluding the study by Du et al. [45]. Similarly, for MSI-H, the pCR proportion ranged from 14% (excluding the study by Wu et al. [46]) to 25% (excluding the study by Du et al. [45]) (Figure S1D). Sensitivity analysis for the risk of not achieving pCR showed no significant variability for both *KRAS* and microsatellite status (Figure S2).

To evaluate a possible confounding due to cancer stage, analyses for pCR by *KRAS* status were further restricted to studies performed in patients at stage II–III LARC [32,33,34,35,37,38]. The summary percentage of pCR was 21% (95% CI: 14–31%) for *KRAS* wild-type and 14% (95% CI: 10–19%; *p* = 0.11) for *KRAS* mutated. Among patients receiving cetuximab, pCR was achieved in 27% (95% CI: 17–40%) of *KRAS* wild-type patients and in 14% (95% CI: 8–23%; *p* = 0.02) of *KRAS* mutated patients, confirming the results of the main analysis.

No publication bias was detected in any analyses (Figure S3).

## 4. Discussion

The possibility to identify good and poor responders in advance for neoadjuvant treatment is a crucial issue in the management of LARC patients, as it could help clinicians select the most appropriate personalized strategy, including intensified pre-operative therapy (e.g., TNT) and organ-preserving approaches [3,9].

Somatic mutations in specific oncogenes (i.e., *RAS, TP53, BRAF, PIK3CA, SMAD4*) and MSI status have been widely studied as predictive markers of response (i.e., pCR) to neoadjuvant chemotherapy or radiotherapy, but the results are difficult to interpret due to the large heterogeneity of the studies performed [18,19]. With this meta-analysis, we attempted to overcome this issue by setting strict inclusion criteria to limit the analysis to a homogeneous study population and avoid potential bias.

This meta-analysis highlighted a significant detrimental role of the *KRAS* mutation, which was found to be predictive of poor response to neo-adjuvant treatment in LARC patients. *KRAS* is a key molecule in the MAPK and PI3K/AKT signaling pathways, which play important roles in cellular differentiation and apoptosis [18]. In colon cancer, somatic *KRAS* mutations, located mainly in codon 12 and 13 of exon 2, have been reported to lead to a more aggressive and invasive tumor and have been associated with resistance to anti-epidermal growth factor receptor (EGFR) monoclonal antibodies such as cetuximab and panitumumab [18,55]. Although the clinical significance of *KRAS* mutations in colon cancer is well established [56], the role of the same mutations in rectal cancer has not been fully elucidated. Pre-clinical investigations have shown that *KRAS* mutations could cause not only a more aggressive tumor phenotype, but also resistance to radiotherapy in rectal cancer [57,58,59]. However, clinical studies attempting to replicate this observation in patients receiving neoadjuvant treatment have been heterogeneous, and no consensus has been reached. Indeed, some analyses have highlighted a possible role of the *KRAS* mutation in predicting a lower pCR rate [32,33], but these results have not been confirmed by other studies [31,35,37]. Our meta-analysis showed that in a group of ten studies that met the inclusion criteria [29,31,32,33,34,35,36,37,38,39], *KRAS* mutations conferred an increased risk of not responding to neoadjuvant treatment (i.e., no-pCR), which is consistent with pre-clinical observations in rectal cancer and the well-established detrimental impact of *KRAS* mutations on the behavior of other tumors. This finding is in contrast to the results of two previously published meta-analyses [60,61], which found no significant association between *KRAS* status and pCR rate. The less stringent inclusion criteria adopted by the two previous meta-analyses could likely account for the discrepancy in results. In addition, one of the two papers [60] was published ten years ago and did not include the most recent data obtained with next-generation genotyping techniques.

During the years 2011–2013, the inclusion of cetuximab in preoperative treatment regimes in LARC patients was investigated. In this context, cetuximab was administered to all patients, regardless of *KRAS* mutational status. Therefore, a subgroup analysis on the predictive role of *KRAS* according to cetuximab administration was possible. The *KRAS* mutation was significantly predictive of pCR only in the group of patients who did not receive cetuximab, whereas it was not associated with pCR in patients who received cetuximab. EGFR has been shown to be a key molecule in the pathogenesis of rectal cancer, and its expression in the tumor of LARC patients undergoing neoadjuvant therapy was associated with significantly lower DFS [36,38]. Moreover, a radiosensitizing effect of anti-EGFR agents was noticed [34,36,37,38]. It could be hypothesized that an interaction between EGFR signaling pathway and radio/chemotherapy is the basis for the observed effect in patients not receiving an anti-EGFR agent such as cetuximab. On the other hand, the specific architecture of the rectal cancer molecular background may have disrupted the interaction between the use of cetuximab and the *KRAS* mutation observed in colon cancer, resulting in no effect of the mutation on the response to the specific anti-EGFR drug [34,36,37,38].

The effect of the *KRAS* mutation on T or N downstaging has also been investigated, but without significant results. This could probably be due to the small number of eligible studies (range 4 to 1), the heterogeneity in preoperative treatment (e.g., studies including cetuximab), and the different endpoints.

In the present meta-analysis, MSI status was not found to be predictive of pCR after neoadjuvant chemoradiation or radiation therapy in LARC patients. This finding confirms the results of two previous meta-analyses, in which a pooled analysis showed no significant effect of MSI status on pCR rates [19,62]. Accumulating data point out a resistance to 5-fluorouracil-based chemotherapy in colorectal cancer patients with MSI tumors [19,62,63]. However, although the MSI status has been reported as a predictive factor for the benefit of adjuvant fluoropyrimidine-based chemotherapy and overall prognosis in colon cancer [64], this finding has not been confirmed in rectal cancer, where MSI leads to different molecular and clinicopathological characteristics than colon MSI tumors [65]. Moreover, an interaction between chemoradiotherapy and MSI may also support our findings. Indeed, chemotherapy and radiotherapy have been reported to reprogram the tumor microenvironment and induce immunostimulatory effects, possibly by promoting a tumor antigen-specific immune response [66,67]. Similarly, MSI status has been shown to alter the radiosensitivity [68,69,70] and influence immunological status during nCRT for rectal cancer [71,72]. Therefore, both chemoradiotherapy and MSI status could affect the immune response, leading to unpredictable outcomes [72]. Our meta-analysis showed a non-significant trend for a lower risk of non-response to therapy in MSI-H tumors, suggesting a positive interaction between the immunomodulatory effect of radiotherapy and MSI status. However, the limited number of studies included in our meta-analysis and the relatively low incidence of MSI in rectal cancer may have compromised the power of the analysis, and further well-designed studies with large samples are needed to definitively clarify whether MSI status can be used to select patients for neoadjuvant treatment in rectal cancer.

Quantitative analysis of the role of *KRAS* mutation and MSI status on recurrence risk and survival was not possible. The literature search revealed that KRAS or MSI status did not appear to have a significant impact on the risk of local recurrence or distant metastasis. Subgroup analyses, e.g., by clinicopathological features (e.g., tumor stage [45]) or molecular features (e.g., specific KRAS codon mutations [29]), could potentially reveal an overlooked association. Moreover, a polygenic risk score that takes into account the interplay of multiple signaling pathways (e.g., the mutated *KRAS* gene and concomitant high expression of vascular endothelial growth factor) could likely better capture the tumor recurrence phenotype [42].

Meta-analysis of the predictive/prognostic value of the somatic mutation in the other oncogenes studied (i.e., *TP53, BRAF, PIK3CA,* and *SMAD4*) was not possible because the minimum number of three eligible articles required for quantitative synthesis of the data was not reached for both response and survival assessment. The gene most frequently studied in this context was *TP53*, with available studies reporting nonsignificant results of association with outcome in LARC patients, both in terms of pCR rate [32,51] and tumor downstaging [51,54]. No eligible studies on the impact of *TP53* mutational status on recurrence risk or survival were found in the literature. Another gene examined in the present meta-analysis is *PIK3CA;* its mutational status was associated with a lack of pCR in one eligible study [52], but this result was not confirmed in another study [53]. The effect of *PIK3CA* mutation on DFS [30] and OS [30] was analyzed by one eligible study without finding a significant association. For the *BRAF* and *SMAD4* genes, no eligible studies were detected for both pCR and prognosis, and the clinical value of their mutational status remains to be investigated.

This study has some limitations. First, there is high heterogeneity among the available studies. A thorough cleaning was performed to select a homogeneous group of studies eligible for meta-analysis. To this end, only studies with molecular data obtained from pre-treatment samples were included in the meta-analysis [70,73]. Two large population-based studies [40,49] were excluded because they collected data from national oncologic databases, and this register-based exploration did not allow the collection of all the information necessary to verify that all eligible criteria, including the timing of molecular analysis, were met. The work of Hasan et al. [49] has also been the subject of criticism of the methodology [62,70]. Nevertheless, a high degree of heterogeneity, including differences in study design (retrospective versus prospective), preoperative treatment (e.g., chemoradiotherapy, intensified chemoradiotherapy, or radiotherapy alone; administration of other drugs in addition to fluoropyrimidines; radiation dose), techniques used for molecular testing, and TRG classification system used to classify response, may still have influenced our work. Second, there are notable differences in the frequency of *KRAS* mutation (range 48–13%) or MSI-H status (range 4–20%) and pCR rate (range 7–43%) between studies, which may reflect the inhomogeneity of molecular techniques across laboratories and over time, as well as different clinical procedures for tumor response assessment or different treatment protocols. However, these differences were accounted for in the statistical procedure chosen. Third, despite some preliminary evidence for a role of the specific *KRAS* mutation (i.e., codon 12, 13, or 146) in tumor phenotype, the limited number of studies on this topic did not allow us to examine the association between specific mutation sites of the *KRAS* gene and treatment response.

## 5. Conclusions

In conclusion, the present meta-analysis provides evidence for the predictive role of tumor *KRAS* mutation in predicting the likelihood of achieving pCR in LARC patients, thus promoting the introduction of a pre-treatment molecular testing to improve the definition of individual risk of treatment failure and guide therapeutic planning. This could translate into personalized treatment, allowing a selective, safe, and organ-sparing approach (e.g., watch-and-wait strategy) in patients with a high probability of pCR and alternative treatment strategies (e.g., total neoadjuvant therapy) in patients with unresponsive tumors. This could lead to fewer surgery-related complications, better quality of life, and fewer unnecessary treatments.

On the contrary, the role of MSI status in predicting response to preoperative treatment remains unclear, and future studies are needed to definitively define its clinical value in rectal cancer. Further research efforts are also required to clarify the predictive significance of somatic mutations in other key oncogenes, such as *TP53, BRAF, PIK3CA,* and *SMAD4.*

## Figures and Tables

**Table 1:** Main characteristics of included studies for *KRAS* gene.

First Author, Year	Country	N	Therapy Strategy	FLs	Other Drug	KRAS Mut (%)	pCR (%)	NOS Score
El Otmani, 2020 [29]	Morocco	57	CRT/RT + surgery	5-FU	-	28%	12%	7
Chow, 2016 [32]	USA	229	CRT/intensified CRT + surgery	5-FU	OXA	42%	26%	7
Duldulao, 2013 [33]	USA	148	CRT/intensified CRT + surgery	5-FU	OXA	41%	25%	7
Sun, 2012 [38]	China	63	CRT + surgery	CAPE	CTX	30%	13%	7
Kim, 2011 [37]	Korea	38	CRT + surgery	CAPE	CTX, IRI	13%	21%	7
Hu-Lieskovan, 2011 [36]	Europe	86	CRT + surgery	5-FU, CAPE	CTX, OXA	40%	12%	7
Erben, 2011 [34]	Europe	57	intensified CRT + surgery	CAPE	CTX, IRI	32%	11%	7
Bengala, 2010 [31]	Europe	141	CRT + surgery	5-FU, CAPE	OXA	19%	15%	7
Zauber, 2009 [39]	Europe	53	CRT/RT + surgery	5-FU	--	34%	43%	7
Gaedcke, 2010 [35]	Europe	93	CRT + surgery	5-FU	OXA	48%	13%	7

Abbreviation: 5-FU, 5-fluorouracil; CAPE, capecitabine; CRT, chemoradiotherapy; CTX, cetuximab; FLs, Fluoropyrimidines; IRI, irinotecan; NOS, Newcastle–Ottawa Scale; OXA, oxaliplatin; pCR, pathological complete response; RT, radiotherapy.

**Table 2:** Pooled percentage of downstaging according to *KRAS* mutational status.

	Downstaging		T Downstaging		N Downstaging	
	Rate (95% CI)	P_Het_	Rate (95% CI)	P_Het_	Rate (95% CI)	P_Het_
Studies (n)	4		3		1	
*KRAS*						
Wild-type	0.52 (0.27–0.77)	*p* < 0.01	0.54 (0.46–0.61)	*p* = 0.48	0.61 (0.49–0.72)	-
Mutated	0.55 (0.37–0.71)	*p* = 0.02	0.44 (0.34–0.54)	*p* = 0.62	0.62 (0.41–0.80)	-
	*p* = 0.87		*p* = 0.14		*p* = 1.00	

**Table 3:** Main characteristics of included studies for microsatellite instability (MSI) status.

First Author, Year	Country	N	Therapy Strategy	FLs	Other Drug	MSI-H (%)	pCR (%)	NOS Score
Wu, 2022 [46]	China	150	CRT + surgery	5-FU	OXA	20%	23%	7
El Otmani, 2020 [29]	Morocco	57	CRT/RT + surgery	5-FU	--	19%	12%	7
Yilmaz Rakici, 2019 [47]	Turkey	37	CRT/RT + surgery	5-FU, CAPE	--	11%	19%	9
Du, 2013 [45]	China	316	RT + surgery	--	--	8%	7%	7
Zauber, 2009 [39]	USA	53	CRT/RT + surgery	5-FU	--	4%	43%	7

Abbreviation: 5-FU, 5-fluorouracil; CAPE, capecitabine; CRT, chemoradiotherapy; FLs, Fluoropyrimidines; MSI-H, high-frequency; NOS, Newcastle–Ottawa Scale; OXA, oxaliplatin; pCR, pathological complete response; RT, radiotherapy.

## Supplementary Materials

The following supporting information can be downloaded at: https://www.mdpi.com/article/10.3390/cancers15051469/s1, Supplementary methods: 1. PICO framework; 2. Search algorithms. Table S1: (A) Characteristics of included studies and (B) details on molecular analysis and response assessment for KRAS gene. Table S2: Quality assessment of included studies for (A) KRAS gene and (B) MSI status. Table S3: (A) Characteristics of included studies and (B) details on molecular analysis and response assessment for microsatellite instability (MSI) status. Figure S1: Influence analyses for percentage of pathological complete response (pCR) according to KRAS mutation and microsatellite status. Figure S2: Influence analyses for the risk of not achieving a pathological complete response (pCR) according to KRAS mutation and microsatellite status. Figure S3: Funnel plots for publication bias.
