# Supplementary material for: Predictive and Prognostic Value of Oncogene Mutations and Microsatellite Instability in Locally-Advanced Rectal Cancer Treated with Neoadjuvant Radiation-Based Therapy: A Systematic Review and Meta-Analysis
Source: Cancers (Basel). 2023 Feb 25;15(5):1469. doi: 10.3390/cancers15051469 (PMC10001009; doi:10.3390/cancers15051469)
Supplement: Supplementary file 1 [file cancers-15-01469-s001.zip › cancers-2113591-supplementary.pdf]

cancers MDPI

Systematic Review

Predictive and Prognostic Value of Oncogene Mutations and Microsatellite Instability in Locally-Advanced Rectal Cancer Treated with Neoadjuvant Radiation-Based Therapy: A Systematic Review and Meta-Analysis

Elena De Mattia, Jerry Polesel, Silvia Mezzalira, Elisa Palazzari, Sara Pollesel, Giuseppe Toffoli and Erika Cecchin

Supplementary methods

1. PICO framework

The PICO framework was used to guide the focus of the systemic review

P (*Patient, Problem, Population*): series including patients with primary adenocarcinoma of the rectum treated with neoadjuvant chemoradiotherapy or radiotherapy.

I (*Intervention*): Have mutation in candidate gene (i.e., *RAS*, *TP53*, *BRAF*, *PIK3CA* and *SMAD4*) or microsatellite-unstable tumor.

C (*Comparison, Control)*: Have no mutations in the candidate gene (i.e., *RAS*, *TP53*, *BRAF*, *PIK3CA* and *SMAD4*) or microsatellite-stable tumor.

O (*Outcome*): Primary endpoint: pathological complete response and tumor downstaging evaluated in accordance with the international guidelines. Secondary endpoint: recurrence risk (i.e., disease-free survival or relapse-free survival) and overall survival.

2. Search algorithms

(chemotherapy OR radiotherapy OR “radiation therapy” OR irradiation OR chemoradiotherapy OR chemoradiation OR chemo-radiotherapy OR radio-chemotherapy) AND ("rectal cancer” OR “rectal carcinoma”) AND (mutation OR mutations OR mutated) AND (RAS OR KRAS OR K-RAS OR NRAS OR N-RAS OR HRAS OR H-RAS OR BRAF OR B-RAF OR PIK3CA OR “phosphoinositide-3-kinase catalytic alpha polypeptide” OR “PIK3 catalytic alpha polypeptide” OR “phosphoinositide-3-kinase” OR SMAD4 OR SMAD-4 OR TP53 OR P53 OR TP-53 OR MMR OR dMMR OR pMMR OR MSI OR “microsatellite instability” OR “replication error” OR “mismatch repair” OR “microsatellite repeats” OR “Genomic instability”)

Table S1. (A) Characteristics of included studies and (**B)** details on molecular analysis and response assessment for *KRAS* gene.

A).

First author, Year	Country	Total patients/ Included for analysis (n=)	Age (Years)	M/F	Study Type	Enrollment interval	Stage TNM	Therapy strategy	RT dose	FLs	Other drug	CRT/RT duration (days)	Interval CRT/RT to surgery (weeks)	NOS score
El Otmani et al., 2020	Morocco	57/57	56 (28-81)	32/25	retrospective	Jan 2012-Oct 2018	na	CRT (n=39) or RT (n=18) + surgery	CRT: 45Gy/ 25 fractions; RT: 39Gy/3 fractions	5-FU	--	35	na	7
Chow et al., 2016	USA	229/229	56 (21-87)	135/94	retrospective	Mar 2004 - Nov 2012	II (n=52), III (n=177)	CRT / intensified CRT + surgery	50.4Gy/28 fractions or 54.0Gy/30 fractions	5-FU	OXA	35-42	4.6-61.4	7
Duldulao et al., 2013	USA	148/148	57 (25-87)	85/63	prospective	na	II-III	CRT / intensified CRT + surgery	504Gy	5-FU	OXA	na	0-16	7
Sun et al., 2012	China	63/63	64 (50–77)	39/24	prospective	Sep 2007 - Mar 2008	II-III	CRT + surgery	45Gy/ 25 fractions	CAPE	CTX	35 RT; 42 CT	6-8	7
Kim et al., 2011	Korea	40/38	56.5 (34–72)	32/8	prospective	May 2006 - Dec 2006	II-III	CRT + surgery	50.4Gy/ 28 fractions	CAPE	CTX, IRI	35 RT; 42 CT	4-8 median: 6.9 (4.4–12.6)	7
Hu-Lieskovan et al., 2011	Europe	130/86	61 (33-83)	74/56	retrospective	na	II (n=4), III (n=109), IV (n=15), na (n=2)	CRT + surgery	50.4Gy/28 fractions (n=42) or 45Gy/25 fractions (n=88)	5-FU (n=16); CAPE (n=114)	CTX, OXA (n=42)	35 RT; 42 CT	4-8	7
Erben et al., 2011	Europe	57/57	57 (33–80)	42/15	prospective	na	II-III	intensified CRT + surgery	50.4Gy	CAPE	CTX, IRI	na	4-6	7
Bengala et al., 2010	Europe	146/141	64 (26–78)	86/60	retrospective	May 1998 - Oct 2005	II (n=59), III (n=83)- IV (n=4)	CRT + surgery	50Gy/ 25 fractions	5-FU (n=132); CAPE (n=14)	OXA (n=34)	35	6-8	7
Zauber et al., 2009	Europe	53/53	65.0 ± 0.5	32/21	retrospective	2002 - 2006	I-II-III	CRT (n=52)/RT (n=1) + surgery	mean dose: 49.12 ± 0,35Gy	5-FU	--	mean 39.2± 4.2	3-16 (delay of 38 weeks for one case); average 8.2 ± 4.86	7
Gaedcke et al., 2010	Europe	94/93	62.3 (35–81)	64/30	prospective	Feb 1995 - Feb 2010	II (n=31), III (n=63)	CRT + surgery	50. Gy/28 fraction	5-FU	OXA (n=37)	35	4-6	7

Abbreviations: 5-FU, 5-fluorouracil; CAPE, capecitabine; CRT, chemoradiotherapy; CTX, cetuximab; FLs, fluoropyrimidines; IRI, irinotecan; NOS, Newcastle–Ottawa Scale; OXA, oxaliplatin; RT, radiotherapy.

B).

First author, Year	Biological Matrix	Mutation tested	Genotyping Method	TRG classification system	*KRAS* mutation (%)	Overall % of pCR
El Otmani et al., 2020	FFPE pre-treatment biopsy	exon 2 codon 12 (G12V, G12D, G12C) and 13 (G13D); exon 4 codon 146 (A146T; A146V)	Sanger Sequencing/Pyrosequencing	Dworak et al.	28%	12%
Chow et al., 2016	FFPE pre-treatment biopsy	exon 2-3, codon 12 (G12V) and 13 (G13D)	Sanger Sequencing/NGS	na^	42%	26%
Duldulao et al., 2013	FFPE pre-treatment biopsy	exon 2 and 3 (codon 6, 12, 13, 22, 61 and 64)	Sanger sequencing	AJCC	41%	25%
Sun et al., 2012	FFPE pre-treatment biopsy	exon 2, codon 12-13	Sanger sequencing	na^^	30%	13%
Kim et al., 2011	FFPE or fresh-frozen pre-treatment biopsy	exon 2, codon 12-13	Sanger sequencing	Dworak et al.	13%	21%
Hu-Lieskovan et al., 2011	FFPE pre-treatment biopsy	exon 2, codon 12-13	PCR-RFLP	Dworak et al.	40%	12%
Erben et al., 2011	FFPE pre-treatment biopsy	exon 2, codon 12-13	Sanger sequencing	JSCCR	32%	11%
Bengala et al., 2010	FFPE pre-treatment biopsy	exon 2, codon 12-14	Sanger sequencing	Dworak et al.	19%	15%
Zauber et al., 2009	FFPE pre-treatment biopsy	exon 2, codon 12-13	Allelic sizes analysis; SSCP	Wheeler et al.	34%	43%
Gaedcke et al., 2010	FFPE pre-treatment biopsy	exon 2, codon 12-13; exon 3, codon 61, exon 4, codon 146.	Sanger sequencing	Gavioli et al.	48%	13%

^ pCR was defined as the absence of tumor cells in the surgical specimen at the primary tumor site and regional lymph nodes. .
^^ pCR was defined as the complete disappearance of all tumor cells.
Abbreviations: AJCC, American Joint Committee on Cancer; JSCCR, Japanese Society for Cancer of the Colon and Rectum; NGS, next-generation sequencing; pCR, pathological complete response; PCR-RFLP, polymerase chain reaction-restriction fragment length polymorphism; SSCP, single-stranded conformation polymorphism; TRG, tumor regression grade.

Table S2. Quality assessment of included studies for **A)** *KRAS* gene and **B)** MSI status.

Study	Selection					Exposure			
	**Is the case definition adequate?**	Representativeness of the cases	Selection of Controls	Definition of Controls	Comparability*	Ascertainment of exposure	Same method of ascertainment for cases and controls	Non-Response rate	Total Score (0-9)
**A)**									
**El Otmani I,2020**	●	●	●	●	○○	●	●	●	7
**Chow OS, 2016**	●	●	●	●	○○	●	●	●	7
**Duldulao MP, 2013**	●	●	●	●	○○	●	●	●	7
**Sun PL, 2012**	●	●	●	●	○○	●	●	●	7
**Kim SY, 2011**	●	●	●	●	○○	●	●	●	7
**Hu-Lieskovan S, 2011**	●	●	●	●	○○	●	●	●	7
**Erben P, 2011**	●	●	●	●	○○	●	●	●	7
**Bengala C, 2010**	●	●	●	●	○○	●	●	●	7
**Zauber NP, 2009**	●	●	●	●	○○	●	●	●	7
**Gaedcke J, 2009**	●	●	●	●	○○	●	●	●	7
**B)**									
**Wu Z, 2022**	●	●	●	●	○○	●	●	●	7
**El Otmani I,2020**	●	●	●	●	○○	●	●	●	7
**Rakıcı S Y, 2019**	●	●	●	●	●●	●	●	●	9
**Du C, 2013**	●	●	●	●	○○	●	●	●	7
**Zauber NP, 2009**	●	●	●	●	○○	●	●	●	7

* 0 point for univariate analysis; 1 point for multivariable analyses with sex and age as covariates; 2 points for multivariable analyses with other covariates in addition to sex and age.

Table S3. A) Characteristics of included studies and **B)** details on molecular analysis and response assessment for microsatellite instability (MSI) status.

A).

First author, Year	Country	Total patients/ Included for analysis (n=)	Age (Years)	M/F	Study Type	Enrollment interval	Stage TNM	Therapy strategy	RT dose	FLs	Other drug	CRT/RT duration (days)	Interval CRT/RT to surgery (weeks)	NOS score
Wu et al., 2022	China	854/150	55 (19–80)	611/243	retrospective	Jan 2013 - Dec 2018	II (n=200), III (n=654	CRT (n=420) + surgery	50.0Gy/25 fractions	5-FU	OXA (n=264)	35	na	7
El Otmani et al., 2020	Morocco	57/57	56 (28-81)	32/25	retrospective	Jan 2012 - Oct 2018	na	CRT (n=39) or RT (n=18) + surgery	CRT: 45Gy/ 25 fractions; RT: 39 Gy/3 fractions	5-FU	--	35	na	7
Yilmaz Rakici et al., 2019	Turkey	37/37	60 (27-81)	21/16	retrospective	2013 - 2016	II-III-IV	CRT (n=35) or RT (n=2) + surgery	45 (n=3) - 50.4 (n=34) Gy/25-28 fractions	5-FU (n=7) or CAPE (n=28)	--	41 (median)	8,8 (median)	9
Du et al., 2013	China	316/316	56 (27-79)	177/139	retrospective	Jan 1999 - Jan 2007	II-III	RT + surgery	30Gy /10 fractions	--	--	14	3	7
Zauber et al., 2009	USA	53/53	65.0 ± 0.5	32/21	retrospective	2002 - 2006	I-II-III	CRT (n=52) or RT (n=1) + surgery	mean dose: 49.12 ± 0,35 Gy	5-FU	--	mean 39.2± 4.2	3-16 (delay of 38 weeks for one case); average 8.2 ± 4.86	7

Abbreviations: Abbreviation: 5-FU, 5-fluorouracil; CAPE, capecitabine; CRT, chemoradiotherapy; FLs, fluoropyrimidines; NOS, New-castle–Ottawa Scale; OXA, oxaliplatin; RT, radiotherapy.

B).

First author, Year	Biological Matrix	Mutation tested	Genotyping Method	TRG classification system	*KRAS* mutation (%)	Overall % of pCR
Wu et al., 2022	na	MMR	MLH1, MSH2, MSH6, PMS2	IHC	AJCC	20%
El Otmani et al., 2020	FFPE pre-treatment biopsy	MSI	MLH1, MSH2, MSH6, PMS2	IHC	Dworak et al.	19%
Yilmaz Rakici et al., 2019	FFPE pre-treatment biopsy	MMR	MLH1, MSH2	IHC	Ryan et al.	11%
Du et al., 2013	FFPE pre-treatment biopsy	MSI	BAT-25, BAT-26, NR-21, NR-24, NR-27	Allelic sizes analysis	Ryan et al.	8%
Zauber et al., 2009	FFPE pre-treatment biopsy	MSI	BAT-26	Allelic sizes analysis	Wheeler et al.	4%

^ pCR was defined as the absence of tumor cells in the surgical specimen at the primary tumor site and regional lymph nodes.
^^ pCR was defined as the complete disappearance of all tumor cells.
Abbreviations: AJCC, American Joint Committee on Cancer; IHC, immunohistochemistry; MMR, mismatch repair; MSI, microsatellite Instability; MSI-H, high-frequency MSI; pCR, patho-logical complete response; TRG, tumor regression grade.

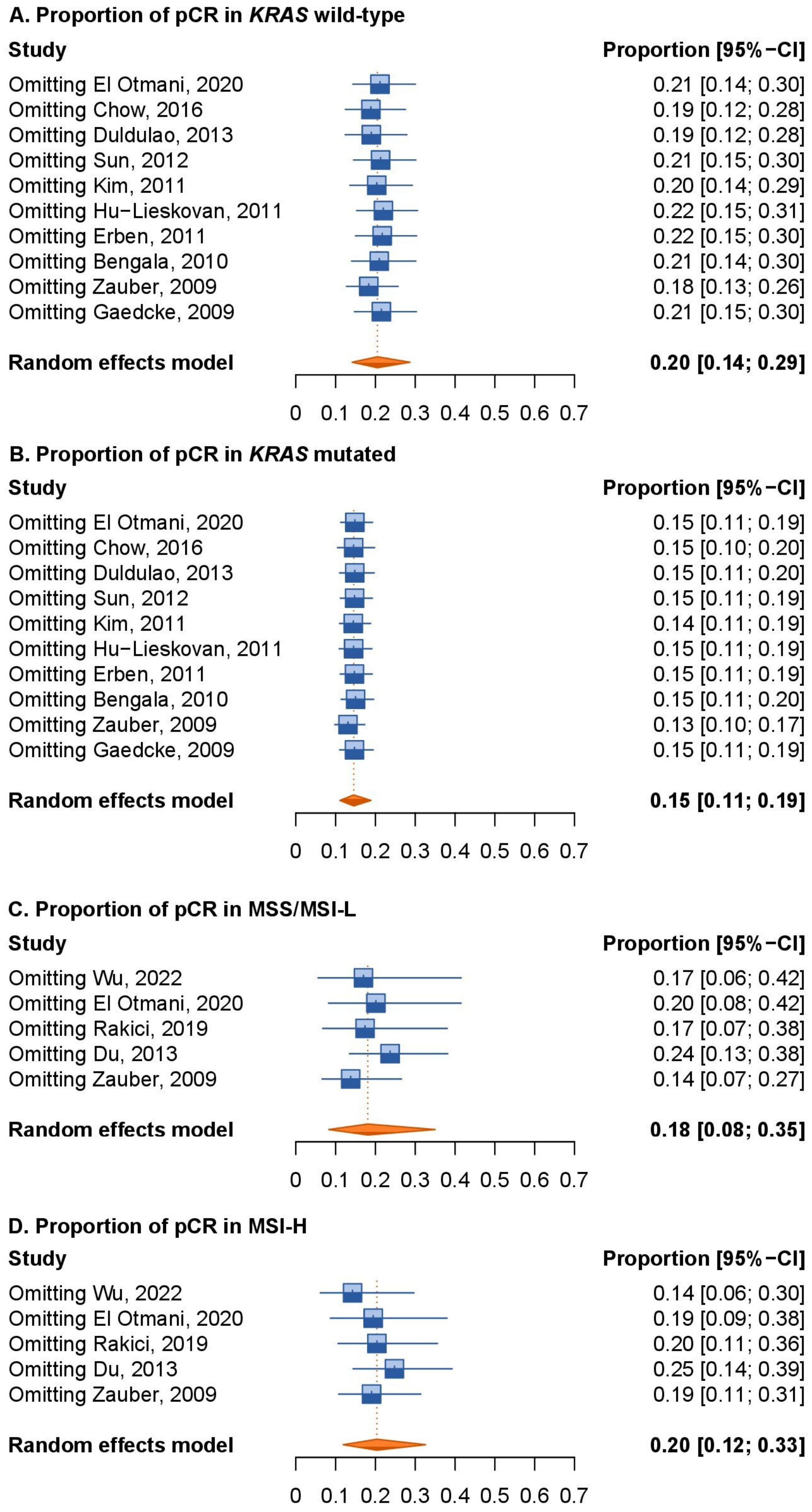

Figure S1. Influence analyses for percentage of pathological complete response (pCR) according to KRAS mutation and microsatellite status.

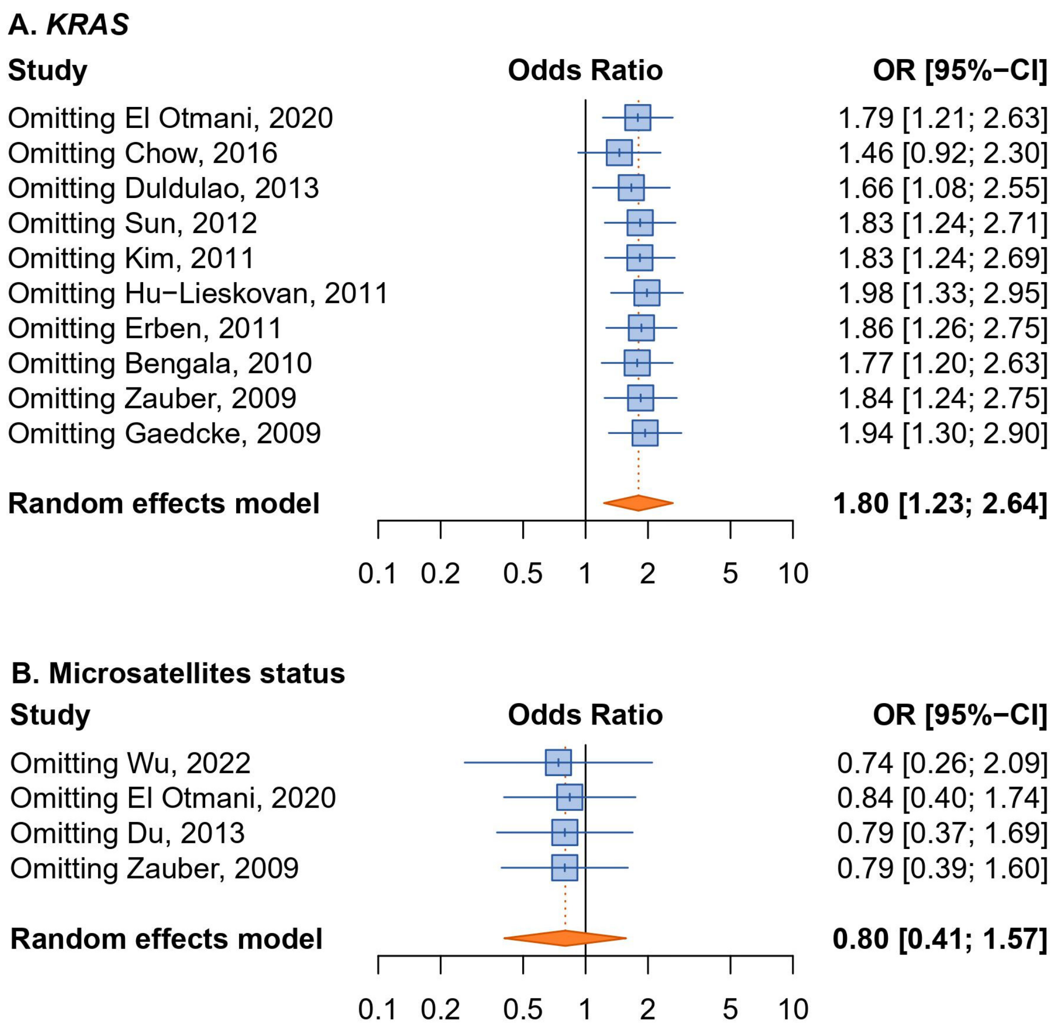

Figure S2. Influence analyses for the risk of not achieving a pathological complete response (pCR) according to KRAS mutation and microsatellite status.

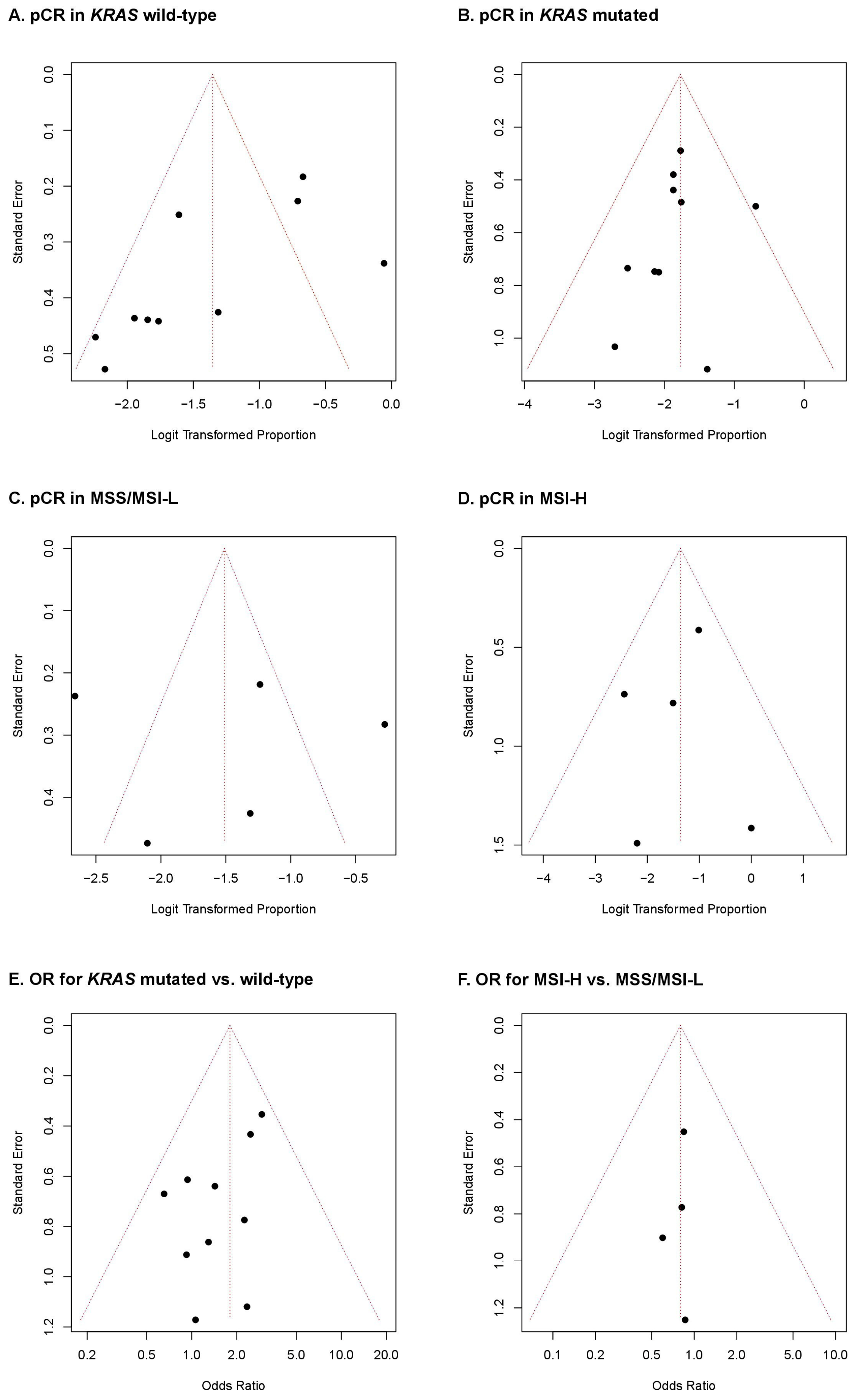

Figure S3. Funnel plots for publication bias.
